# A history under construction: Manguinhos-Maré in the present time

**DOI:** 10.1590/S0104-59702023000100062

**Published:** 2023-11-10

**Authors:** Renato da Gama-Rosa Costa, Renata Soares Costa Santos, Matheus Gonçalves Góes

**Affiliations:** i Pesquisador e professor, Departamento de Patrimônio Histórico/Fundação Oswaldo Cruz. Rio de Janeiro – RJ – Brasil gamarosacosta@gmail.com; ii Pesquisadora, Departamento de Patrimônio Histórico/Fundação Oswaldo Cruz. Rio de Janeiro – RJ – Brasil r.soarescsantos@gmail.com; iii Arquiteto, Departamento de Patrimônio Histórico/Fundação Oswaldo Cruz.Rio de Janeiro – RJ – Brasil arq.matheusgoes@gmail.com

**Keywords:** Fundação Oswaldo Cruz (Fiocruz, Manguinhos-Maré, Present time, Urban history, Fundação Oswaldo Cruz (Fiocruz, Manguinhos-Maré, Tempo presente, História urbana

## Abstract

The two first decades of the twenty-first century were representative of the contemporary history of the Oswaldo Cruz Foundation (Fundação Oswaldo Cruz) and demonstrated its active role in the national health scenario. This article discusses the spatial transformations that took place in the territory occupied by the foundation from the year 2000 onward. The transformations in the use of this territory and it’s institutional policy within the national context are described, along with the social demands that have impacted the institution, namely large-scale urban transformations and pandemics. This research uses the premises of the history of the present to investigate the changes that took place on the Manguinhos-Maré campus and in the discourse of the institutions involved in managing and planning these changes through analysis of institutional reports, digital mapping, and aerial images.

## Resumo

As duas primeiras décadas do século XXI foram representativas para a história contemporânea da Fundação Oswaldo Cruz e demonstraram seu protagonismo no cenário nacional da saúde. O artigo apresenta transformações espaciais ocorridas no território ocupado pela instituição a partir de 2000. Relacionam-se as transformações no uso dos territórios com sua política institucional no contexto nacional e são ressaltadas as demandas sociais que impactaram a instituição – transformações urbanas de grande porte e pandemias. O trabalho averigua, sob os pressupostos da história do tempo presente, as mudanças ocorridas no *campus* Manguinhos-Maré e no discurso de sujeitos institucionais gestores e projetistas. Para isso, conjuga análise dos relatórios institucionais, da base digital cartográfica e de imagens aéreas.

On the centennial of the Oswaldo Cruz Foundation, an exhibition entitled “100 Years of Architecture in Manguinhos” was held at the Brazilian Institute of Architects (Instituto de Arquitetos do Brasil, IAB). A substantial quantity of material was gathered for this exhibition, under the curatorship of the Casa de Oswaldo Cruz’s Department of Historical Heritage and signaled the president of this institution’s interest in further study on architectural production in Manguinhos. This opened the door to a broader survey of the urban evolution of the campus, conducted by the Department of Historical Heritage at the Casa de Oswaldo Cruz (DPH/COC) between 1998 and 2003 and funded by the COC/Fiocruz Special Research Program (PEP). This effort was also justified by the need to provide support for technical requests from the Brazilian National Institute of Historic and Artistic Heritage (Instituto de Patrimônio Histórico e Artístico Nacional, Iphan) to update data on this site of historic interest for federal protection. Documents, oral statements, photographs, and maps were collected and served as the origin for a publication entitled *Um lugar para a ciência: a formação do campus de Manguinhos [A Place for Science: the Shaping of the Manguinhos Campus]* (Oliveira, Costa, Pessoa, 2003).

This study used material from historical archives that was required to survey the history of the institution and the city of Rio de Janeiro, such as documents, institutional reports, maps, newspaper articles, and photographs from a variety of sources beyond the institution’s own collections. Much of this information came from archives outside of Fiocruz, particularly institutions such as the Air Force Cultural Institute (Instituto Cultural da Aeronáutica, Incaer), the Rio de Janeiro Water and Sewage State Company (Companhia Estadual de Águas e Esgotos do Rio de Janeiro, Cedae), the Rio de Janeiro Light and Power Company (Companhia de Energia do Rio de Janeiro, Light), the Rio de Janeiro Regional Council for Architecture and Engineering (Conselho Regional de Arquitetura e Engenharia do Rio de Janeiro, Crea), the *Jornal do Commercio* newspaper, the General Archives of the City of Rio de Janeiro, the Getulio Vargas Foundation Center for Research and Contemporary Documentation (Centro de Pesquisa e Documentação Contemporânea da Fundação Getulio Vargas, CPDoc-FGV), the Brazilian National Archives, the Rio de Janeiro Municipal Urban Cleaning Company (Companhia de Limpeza Urbana do Rio de Janeiro, Comlurb), the Campo dos Afonsos Air and Space Museum, and the Noronha Santos Library (Iphan-RJ).

The methodology utilized studies conducted on “historical sedimentation” and “historical stratification” (Oliveira, Costa, Pessoa, 2003, p.17), and consists of developing maps representing a certain area and using strata or layers to show how it transformed or become established (“sedimentation”) during a certain period of history. This research produced 11 maps that represent the evolution of the Manguinhos site by decades from 1900 to the 2000s.

Nearly twenty years after the publication of the results of the first study, the institution yet again has signaled the importance of recording changes in the Manguinhos campus layout. The current study,^1^ considering the time limits utilized, analyzed the source materials based on reflections offered by the history of the present time. This involved using contemporary sources produced almost simultaneously alongside the research that was being conducted, such as digital materials (texts, reports, and images) and interviews, for example. Sources included institutional reports, annual activity reports, and documents resulting from discussions in internal congresses held at the institution in 2002, 2006, 2012, and 2014.

**Figure 1:**
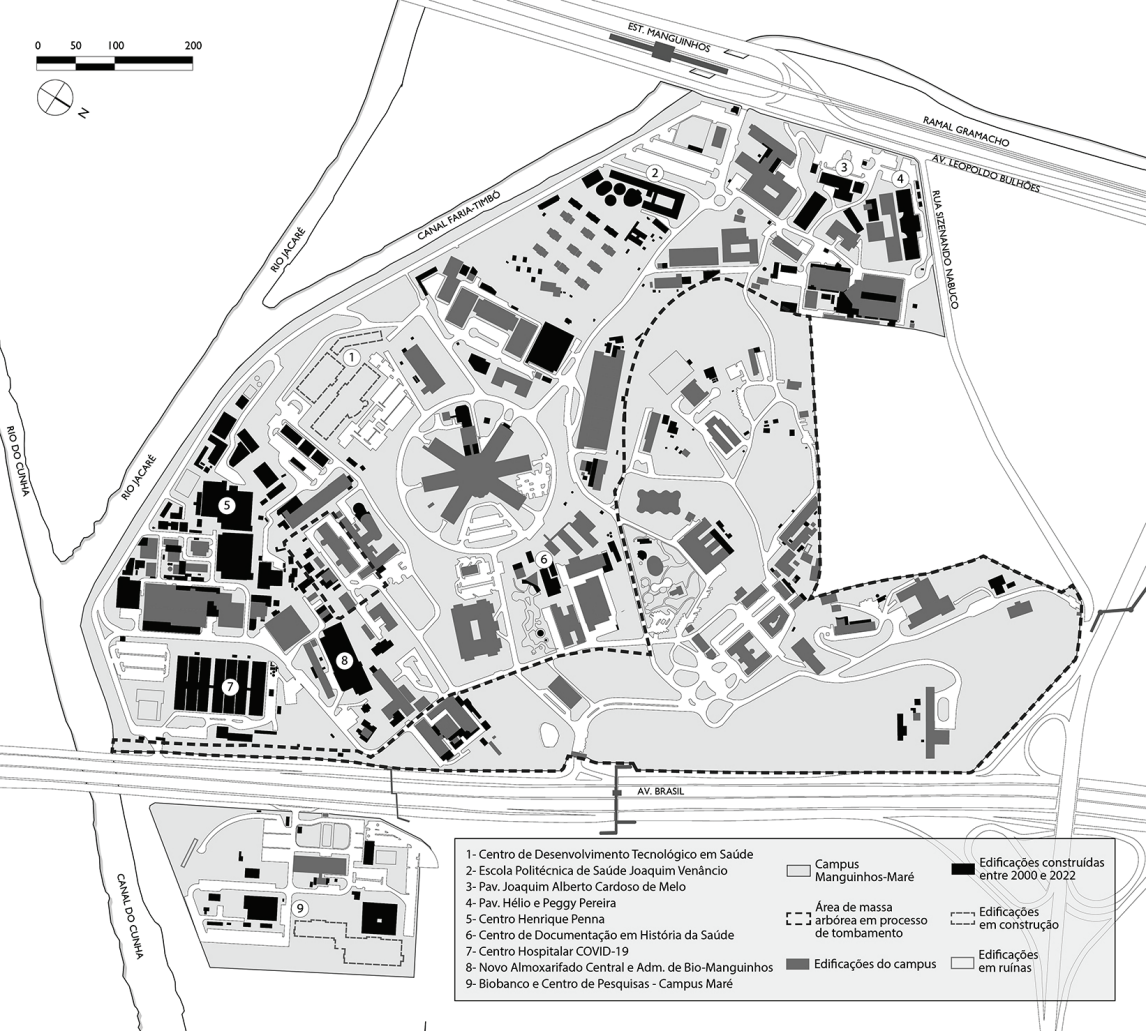
: Map of the Manguinhos-Maré campus (2000-2020) showing the new buildings constructed over the past two decades and identifying those highlighted in the study (DPH/Fiocruz, adapted by the authors)

The methods used in the original study to develop maps were maintained and an additional two maps were created for the 2010s and the 2020s, presented here in a preliminary manner and shown in Figure 1 . The maps featured in the 2003 publication were created using vector design software, based on the *Levantamento Aerofotogramétrico Digital do Município do Rio de Janeiro [Digital Aerial Survey of the Municipality of Rio de Janeiro]* , which was carried out in 1999 by the Pereira Passos Institute (Prefeitura..., 1999). The current maps were developed using a similar program and the same technique based on the set of maps from 2003 recovered from the institution’s archives, recent campus mapping produced by the General Coordination Office for Campus Infrastructure (Coordenação-geral de Infraestrutura dos *Campi* , Cogic), as well as a 2013 aerial survey conducted by the Rio de Janeiro city government (Prefeitura..., 2013) and mosaics of satellite images obtained from Google Earth (Google Earth, 2022).

Maps were used as a methodological resource in order to visualize the findings of the survey and as a possibility for interpreting reality. Separating the maps into temporal layers reflects the evolution and trajectory of the institution as a physical space that involves social experiences, construction, deconstruction, and transformations in this territory. The common understanding of cartography as a simple, neutral, and impartial graphic representation of space certainly does not translate its true meaning, which we can understand to be a graphic representation of a narrative, a story that simultaneously unites man, time, and space ( Oliveira, 2020 ). In this way, cartography can be understood as an interpretation of the world that consequently contains value judgments. It is influenced by all the social issues that surround the object of study, and is subject to the selectivity imposed by the author in the criteria used to omit or show geographic data related to the space. In this way, mapping becomes a partial visual instrument that illustrates a certain territory and its characteristics during a certain period within its historical trajectory ( Harley, 2005 , 2009 ).

The investigation conducted (in the previous as well as the current studies) is not intended to be just an institutional history, although our reflections certainly contribute to such a purpose. Analyzing the main events that took place in the institution and in the city of Rio de Janeiro during the study period helped us interpret the urban transformations and architectonic constructions carried out at Fiocruz in Manguinhos, permitting a systematic reading of the campus’s trajectory over 120 years.

The final decade analyzed in the 2003 publication indicated the start of some projects and construction that were already underway, and others that were in the planning stages or which would solidify in the following years. Many were concluded in the 1990s, which caught the attention of the first study’s authors: “Doubtless the greatest number of accomplishments in the history of the institution” (Oliveira, Costa, Pessoa, 2003, p.193).^2^ Initially, we can see that the 2010s and 2020s were even more significant in terms of accomplishments. The survey identified around 117 completed projects, among smaller facilities or those dedicated to technical or logistical support like energy substations, storage areas, water towers etc. (58), temporary modular structures like containers (19), and large-scale buildings (40).

Some of these projects were analyzed, including the larger ones that brought about greater involvement by the Fiocruz units in terms of innovation and the need to expand and invest in new areas and/or those that were more established in the institution (Relatório final..., 2012). These represent the largest alterations in the makeup of the current Manguinhos campus. So far, the research has analyzed the reasons and negotiations to construct each building: the need, controversies, and context in which they were proposed, as well as the development of the architectural plans, the teams involved, and the implementation of the project on the campus or campuses. These buildings include the headquarters of the Joaquim Venâncio Polytechnic Health School (2004), the Joaquim Alberto Cardoso de Melo Pavilion (2005), the Hélio and Peggy Pereira Pavilion (2001-2008), the Henrique Penna Center (2006), the New Central Warehouse and Bio-Manguinhos Administrative Building (known as the Vinícius Fonseca Administrative Center) (2013-2016), the Center for Documentation in the History of Health, headquarters of the Casa de Oswaldo Cruz (2018), the Covid-19 Hospital Complex (2020), the Health Technology Development Center (under construction), and the installations of the Maré campus (also under construction).^3^

These projects involved at least three design engineering teams, including both architects and engineers: one linked to what used to be Dirac but today is Cogic,^4^ one at Bio-Manguinhos, and one at Casa de Oswaldo Cruz. Separating these projects by what appear to be independent teams could have consequences for the way in which the area at Manguinhos is occupied. We can see on the maps how the spaces near the riverbanks and on the far edges of the campus, outside the protection area demarcated by Iphan in 1989, have exhibited unplanned densification in recent decades, while occupation within the protected area has been preserved to a certain extent.

In general, the implementation of these new buildings calls attention to a trend observed at Manguinhos in the 2000s. As the institution’s activities expanded, locations peripheral to the large buildings along the Faria-Timbó canal and Jacaré river were identified, near the exits to Leopoldo Bulhões street or the delivery entrance on Brasil avenue, or even in the expansion area (which came to be known as the Maré campus), to avoid occupying central areas near the historical spaces.

A large number of container-based structures are also visible, indicating an institutional tendency to meet the emergency needs of the units by installing teams and services in spaces that could be installed quickly and were supposedly temporary but in fact remained on the campus for quite some time. Using this same emergency rationale, a hospital was built to serve the victims of covid-19, one of the institution’s major investments over these past two years. Although we do recognize the importance of studying this hospital, it is not our focus of analysis in this article. We would like to express our concern with the continuity of the hospital at this time, since we know its structure does not permit a long life. The issue is its permanence on the campus, which simultaneously it indicates the slow decommissioning of the former Evandro Chagas Hospital^5^ which dates back to 1918 but at the time of this writing is undergoing transformations in how it is utilized (Costa, Santos, Martire, 2022).

**Figure 2:**
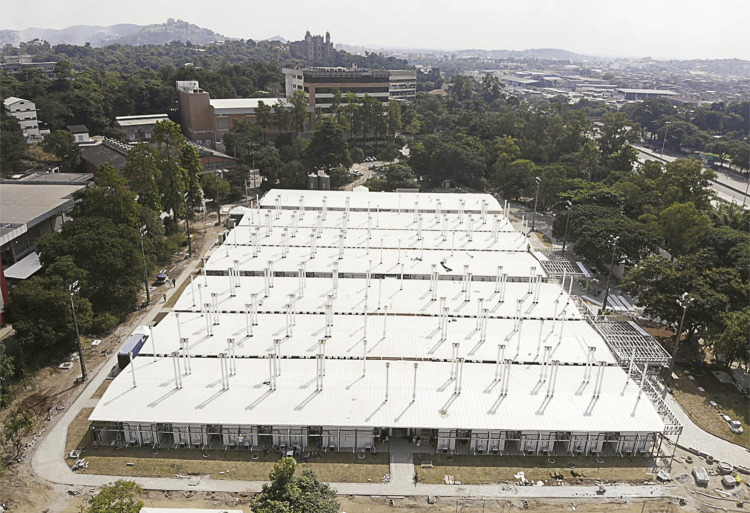
: Aerial image of the Covid-19 Hospital Center. In the background, the building that houses the headquarters of the Oswaldo Cruz Foundation, the Mourisco Pavilion (Asmontec’s archive)

From the aforementioned building survey, we selected two symbolic cases for analysis and reflection on the current situation at the institution: the plan for the Biotechnology Center or the Health Technology Development Center (Centro de Desenvolvimento Tecnológico em Saúde, CDTS), and the buildings that comprise the new Maré campus. We shall explore both as examples of an “unfinished history,” a concept that is held dear during current debates and which generally positions us amid the limitations of a historical analysis of a time that has not yet concluded or imposes a significant time lag to obtain historiographic consensus. This is because interpreting the facts and discourses of the actors involved and the negotiations becomes even more complex when everything is still under construction. The first building, the CDTS, represents the investments Fiocruz has been making in biotechnology, in this case since the 1990s and more assertively since the mid-2000s. The project spans nearly forty years of scientific innovation at the institution and has not yet concluded. Meanwhile, the new facilities at the Maré campus were initiated more recently; the Covid-19 Biobank (BC19-Fiocruz), the Biological Resource Center, and the Center for Research, Innovation, and Vigilance in Covid-19 and Health Emergencies were stimulated by the challenges stemming from the pandemic and are intended to support research on new pandemics in the future.

## Manguinhos in the present time

Studies of the present return to the historiographic concerns expressed during the second half of the twentieth century with expanding validation in the field and methods capable of generating reflection and writing. These studies were validated in various European countries, at first most notably in France, and this process was marked by internal resistance, since recent history had been interpreted as a problematic subject in the field of history (Delgado, Ferreira, 2013; Dosse, 2009 ; Ferreira, 2018 , 2000 ; Hobsbawm, 1993 , 2004 ; Kaelble, 1993 ; Lohn, Campos, ago. 2017; Arend, Macedo, 2009).

Ferreira (2000 , p.111) states that the history of recent facts was not always seen this way; on the contrary, he recalls that during Antiquity it was a central point in the eyes of historians, since “history was a repository of examples that should be preserved, and the work of the historian was to expose recent facts attested to by those who witnessed them directly” (p.11). Direct witnesses were valuable sources and real boons for historical studies, but lost relevance after the institutionalization of history as a discipline in the nineteenth century, which in turn granted more legitimacy to temporal distancing and analysis of written sources as a condition for an interpretation of history, solidifying the triumph of a concept of “scientific” history. This conception offers another relationship between the historian and the document as a research source, which necessarily requires temporal distancing and the historical decision to archive or catalog it as a source. On the other hand, studies of the present time add another dimension to the notion of the document which now includes new possibilities for historical interpretation: oral accounts, interaction with social media, fieldwork, and the involvement and personal experiences of the researchers.

Expressions like *histoire du temps présent* , contemporary history, and *Zeitgeschichte* were integrated into the vocabulary of historians after Second World War ( Kaelble, 1993 ), and interest has only grown since that time. But there is still no consensus among historians about the expression “present time.” Still, over recent years studies that expanded and legitimized fieldwork have gained strength against resistance to incorporating them as objects. These studies do not ignore the difficulties, but rather show the possibilities of writing from the present in the field of history (as the main references used in the article).

This work follows in the footsteps of these studies and contributes to the writing of the contemporary history of Fiocruz, with an eye to the present moment and to recent years in light of the events that took place at the institution where we, the researchers, are located. In this article, aware that no historian can ever escape questions about his or her own time ( Rousso, 2006 ), and in line with the position taken by Lohn and Campos (ago. 2017), we examine some possibilities created by the history of the present time as both a conceptual reflection as well as a methodological attitude.

Ferreira (2018) posited that one orientation should prevail in defining the present time: the presence of individuals who played active roles in or witnessed the past. These individuals are responsible for offering their reports and narratives as historical sources, which are extremely valuable for the historian’s analysis. This concept lines up perfectly with those offered by Ricoeur (2007) , in emphasizing that the history of the present time is “that in which the words of still-living witnesses come up against the writing that contains the documentary traces of the events considered” (p.456). Along these lines, by valuing oral reports, we opted to analyze the narratives of individuals who played important roles in the new buildings on the Manguinhos campus over these past two decades at the institution. And we made these efforts to write history while facing one of the greatest difficulties of narrative about the past in the present time: the different perceptions that intersect in the same objects of study, and the involvement of the researchers themselves.

## Health Technology Development Center

During its institutional life, the Oswaldo Cruz Foundation has faced a series of challenges related to public health in Brazil. Since the 1990s it has invested in innovation strategies in the area of biotechnology, and more specifically, since 2005 it has been constructing the Health Technology Development Center (CDTS). The covid-19 pandemic accelerated some strategies, such as investments in vaccine manufacturing and the construction of a new hospital complex to treat victims of this illness. The establishment of the Biobank in the Biological Resource Center (Centro de Recursos Biológicos, CRB) and the Center for Research, Innovation, and Vigilance in Covid-19 and Health Emergencies were also part of this institutional effort to face this pandemic and others that may follow it, marking the 2020s as one of the most important decades in the history of this institution.

The CDTS is one of the buildings pointed out in the 2003 publication as having its beginnings in the late 1980s and early 1990s. It was originally conceived during the term of Sérgio Arouca (1985-1989) as president of Fiocruz as a Center for Biotechnology. But according to Morel (27 mar. 2019), Arouca was unable to obtain the resources needed at the time from the government of then-president Fernando Collor de Mello, and had to wait 15 years to return to the project and for its construction to be approved during the institution’s fourth Internal Congress held in November 2002.^6^

In order to explore the history of this building on the campus, we analyzed institutional records at Fiocruz such as reports from the internal congresses, but we also needed to face one of the challenges of writing about “our own time,” using experiences with field visits and oral accounts as a resource. The working group visited the 15,000m^2^of the building, which was still under construction, which inevitably intertwined our narrative of the experience of walking the building’s five stories with what we heard from the professionals involved in the project. Between this listening and observing the reports, we were able to understand the main questions that guided the CDTS project throughout its construction and put together the puzzle pieces of this history, at the time of the field visit as well as during the interview held at the DPH.

As for the unique nature of the reports, Rousso (2006) points out that as sources of information about an individual’s experience, they are inaccessible by archives due to their contemporaneity. And despite their documentary richness, “this ‘source’ is no more or less important for historians who address the history of remembering an event than for those who address the event itself” (p.98).

In this way, interpreting the CDTS project as a historical happening through these sources speaks to the concept of the significant to Ricoeur (1997 , p.243) in terms of historical events, about which he stressed that “it is not just what happens, but what can be told or what has already been told in the chronicles or legends.” This narrative unquestionably occurs in the present, as maintained by the historian Marc Bloch (2001) in emphasizing that history is not the study of the past, but rather a way of getting to know the past, and that this knowledge is modified and transformed through the concerns of the time at which the knowledge was produced. This discussion was later expanded by Le Goff (1990 , p.51), another French historian linked to the *Annales* school,^7^ who called attention to the fact that “all history is quite contemporary to the extent that the past is understood in the present and consequently responds to its interests, which is not only inevitable but legitimate.”

About the early concerns of the CDTS project, Carlos Morel, a researcher at Fiocruz and coordinator of the CDTS, highlights that Fiocruz felt the need to update its technological complex, which was considered outdated in terms of technological development and innovation, investing in the production of vaccines, pharmaceuticals, and diagnostic kits (Morel, 27 mar. 2019; Costa, 2019 ). This interest in a biotechnology center was profoundly aligned with research linked to researchers at the Oswaldo Cruz Institute that stirred global interest in molecular biology (Galler, 2005). It was intended to connect basic research and production, traditional areas of the institution, with technological development and applied research, connecting public and private investments: “The creation of the Center represented an innovation for Fiocruz in relation to its activity, since it addresses a specific research niche, as well as with regard to the form of organization” ( Costa, 2019 , p.105).

With arguments about achieving national sovereignty in the area of technological development and guaranteeing investments to combat neglected diseases, the project required major investment by the institution, which needed to create facilities from the ground up and not adapt any existing buildings. The construction needed to keep up as the project evolved (the plan was revised five times) and involved a number of hurdles, and even today the building is not yet fully functional; according to Rodrigo Costa (2019 , p.103, 2022), “construction is not yet concluded and its plan is still being revised.”

After the Federal Public Audit Office gave final approval to start the project in July 2008, the Campus Administrative Directorship (Dirac) asked to supervise it and determined that the entire project needed to be reviewed, after having become technologically outdated in the few years between delivery of the plan and approval by the Federal Public Audit Office. This revision meant canceling the construction work after March 2012 until the required modifications could be addressed and all the administrative and contractual squabbles could be resolved. It only resumed in May of the following year, at which point responsibility passed to Bio-Manguinhos, which made its own changes to the project, causing additional delay. This placed the onus for continuing the project back with Dirac. And in November 2015, a new internal congress decided that the project would definitively become the responsibility of Fiocruz’s vice president for Management and Institutional Development ( Costa, 2019 ; Fiocruz, 31 mar. 2016). During this event, the CDTS was granted the competency to conduct reference activities in translational science to develop health products and processes. Work resumed in December 2018. A new version of the project has been under construction since 2019 and is still not complete.

The plan consists of three buildings: the main one, containing approximately 15,000m^2^and five floors, the animal experimentation space, with three stories and 4,000 m^2^, and the utility building, spanning one floor and 1,000 m^2^, with a total area of 20,000 m^2^. The site chosen for the installation was a portion of the hill near where the Faria-Timbó and Jacaré rivers meet. The main facade faces the building that serves as the headquarters for Fiocruz, the Mourisco castle, in order to take advantage of this view, as explained by Rodrigo Costa (2022) in his interview with the team and confirmed during the visit to the site that same year. The main building houses the laboratories and the administrative and support spaces. The utility building holds most of the central machinery that is required for the functioning of the other two buildings. The main building is the largest of the three in terms of height, length, and volume; it concentrates the most activities and serves to organize the other two peripheral buildings, with 75% of the total constructed area. More generous apertures with plenty of windows were planned for this main building, while the animal experimentation space and utility building are more closed, with smaller openings, particularly on the southern sides where the Manguinhos Favela is located ( Costa, 2019 ). This is also true for the roof, where the highlight is the curved element of the main building.

**Figure 3:**
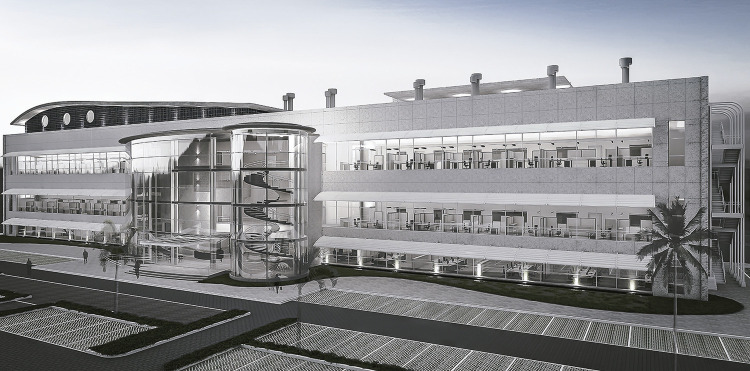
: Main facade of the Health Technology Development Center Center (Paal Architecture’s Archive)

## Maré campus

The discussion about the Maré campus sets us squarely in front of one of the largest hurdles in writing about the present time: writing about something that is taking place at the same time as the research. This requires reinforcing that the history of the present time is an incomplete history by nature, “a history in constant movement, reflecting the commotions that unfold within us and consequently the subject of unending renovation” ( Bédarida, 2006 , p.229). Hobsbawm (2004 p.11-26) says that despite the structural problems in the history of the present time, it is still necessary. He states that there is no choice, and again reinforces the need to conduct research with the same care and the same criteria as for other time periods, even if it is just to save the sources that will be indispensable for historians in the third millennium from being forgotten or even destroyed. In this way, convinced of the importance of presenting another significant building at Fiocruz from a historical point of view, this article examines the Maré campus.

This campus that locates an old Health Station building (which belonged to the Ministry of Health at that time, constructed in the 1970s) and also previously known as the “expansion” of the Manguinhos campus, was donated to the institution by the federal government for installation of technical and administrative units of Fiocruz on September 21 (Brasil, 23 set. 2021); to a certain degree, it recovered the land where the old Federal Sorotherapy Institute^8^ was located prior to the construction of Brasil avenue (1939-1954). It comprises land and riverside areas spanning 58,205,50m^2^located at number 4036 Brasil avenue, opposite the Manguinhos campus.

As seen in the final page of the 2003 publication, the intention was to construct a new set of buildings during the era known as the Niemeyer Complex (Oliveira, Costa, Pessoa, 2003, p.200-203). The plan, authored by Oscar Niemeyer, involved five new pavilions on the space where the former Health Station was located (today the Maré campus), as well as in the old soccer field that today is occupied by the Covid-19 Hospital Complex on the Manguinhos campus: the Museum of Microscopy, the Exposition Pavilion, a convention center, a service pavilion, and the Life Observatory. But this set of installations ultimately was not constructed, possibly due to institutional choices that resulted in the construction of various other buildings over the past two decades. Meanwhile the integration of the two areas belonging to Fiocruz in Manguinhos, separated by Brasil avenue, which involved an overpass, is still considered necessary by the institution. The plan is under study, under the title “Science Overpass.”

The Maré campus is comprised of the main building (formerly the building where the 15th Federal Health Station was located), a sports/recreation area, the Fiotec building, and a set of facilities built during the covid-19 pandemic: the Covid-19 Biobank (BC19-Fiocruz), opened in December of 2021 (Fiocruz inaugura..., 13 dez. 2021), the Center for Biological Resources, and the Center for Research, Innovation, and Vigilance in Covid-19 and Health Emergencies, which is still in the final stages at the time of this writing, as well as areas with archaeological potential.

The Biobank is “appropriate infrastructure for the safe, reliable, ethical, legal, and traceable storage of human and non-human (virus) samples related to covid-19 and to the SARS-Cov-2 virus and variants of interest for research, technological developments, and innovation” (Do CRB-Saúde..., s.d.). According to Fiocruz President Nísia Trindade Lima,^9^ the construction of the Biobank is in line with the mission of the institution, “by helping to overcome the crisis caused by the covid-19 pandemic, a building that will contribute much to our Brazilian Unified Heath System (Sistema Único de Saúde, SUS)” (Fiocruz inaugura..., 13 dez. 2021).

BC19-Fiocruz, a 1,100m^2^facility, was constructed with funding from the Ministry of Health intended to create centers that permit the design and execution of studies and clinical trials related to covid-19, regardless of where the biological specimens were collected. According to the institution, the Biobank’s collections consist of human biological samples and “will be capable of storing approximately 1.5 million samples under appropriate conditions ... including an automated system for collection storage” (Do CRB-Saúde..., s.d.).

Meanwhile, the Biological Resource Center (Centro de Recursos Biológicos, CRB) preserves a collection of viruses, comprising “cultivatable organisms, and replicable parts thereof, as well as databanks containing molecular, physiological, and structural information associated with these collections” (Do CRB-Saúde..., s.d.). Both comprise a platform which is open to scientific and technical institutions, fostering collaborative efforts at the domestic and international levels.

According to an institutional report, since 2005 Fiocruz has invested in structuring a Center for Biological Resources in Health as a way of establishing a collection of cultures of pathogenic microorganisms whose origins are related mainly to tropical diseases or those with biological potential in the area of health care. The report does not mention it, but certainly this investment has been made since the construction of the Health Technology Development Center, leading to the question: how will the institution connect these two projects, establishing the CDT *S* and the facilities that comprise the new Maré campus? According to the same report, the CRB-Saúde was intended to contribute to and support research, technical development, and innovation by offering certified products and services to the scientific community and to the SUS, provide high-quality products and services to develop diagnostics, vaccines, and medications in accordance with legal and international requirements for biosafety, protection, and quality, and strengthen the Economic-Industriual Health Complex (Complexo Econômico-Industrial de Saúde, Ceis) in order to reduce Brazil’s international dependence in the area of health innovation (Do CRB-Saúde..., s.d.).

The construction of the Center for Research, Innovation, and Vigilance in Covid-19 and Health Emergencies began in October 2021, while measures to fight the coronavirus such as social distancing and working from home were still in force. The new research center is intended to help face future health emergencies and be part of scientific studies on covid-19, “including the behavior of the virus and response to prophylaxis and immunizations, but also to support a forward-looking structure for other health emergencies” (Fiocruz inicia..., 25 out. 2021). With an estimated timetable for execution of 12 months, under the responsibility of Cogic’s team of architects and engineers, the construction process has altered the urban and social dynamics in the area known as the “Manguinhos campus expansion” and its surroundings, which are composed of single and multi-family dwellings that are part of the Maré district.

These inconveniences, such as noise and air pollution and road detours resulting from the construction, can be perceived in daily life at the institution, but are not easily mapped. One way to observe this issue is reports on social media and messaging applications that offer elements and clues, although they are not sufficient for more definitive conclusions. This apparent vulnerability of construction of knowledge about a certain time, the present, does not rule out a contemporary analysis or interpretation, but rather only reinforces what Paul Ricoeur (1997) noted in his theoretical contributions on the happenings of our own times, understanding them to be part of an open and indeterminate process. In his words, “it is necessary to fight the tendency to consider the past from the point of view of something that is over, unchangeable, unwavering” (p.372).

The scientific and technological complex is composed of buildings that total 13,000m^2^and urbanized area of over 12,000m^2^. Its objective is to gather researchers from different knowledge areas in 15 laboratories optimized for use, according to institutional guidelines proposed at Fiocruz’s seventh Internal Congress, which makes it possible to share “the use of laboratories, equipment, platforms, human resources, research protocols, and intellectual capital” (Fiocruz inicia..., 25 out. 2021), similar to the plan for the new CDTS facilities.

The research and animal experimentation (small rodents) laboratories both are biosafety level 2 and 3 compliant (BSL-2 and BSL-3). The main building, located directly behind the old Health Station building (situated on the edge of the property where there is a wall separating the campus from the rest of the Maré neighborhood), will have three floors; following the same model utilized in the CDTS building, one floor will be exclusively technical in order to permit maintenance with less impact on the research environments. According to the report, the new center will allow Fiocruz to continue “strengthened and even better equipped to serve and conduct research ... by intensifying activities in scientific and technological development which can be translated into products and knowledge that benefit the SUS and the Brazilian population” (Fiocruz inicia..., 25 out. 2021).

The idea in structuring the new Maré campus is to transfer the functions of the Center for Studies on Worker Health and Human Ecology (Centro de Estudos de Saúde do Trabalhador e Ecologia Humana, Cesteh) to the other side of Brasil avenue, and the plan proposed demolishing these facilities along with other services on the Manguinhos campus such as the former polytechnic facilities and the Osório de Almeida Pavilion, which were all built with a modular structure planned by the architect João Filgueiras Lima and hold major significance for the campus. This in fact indicates that the movement of the areas (and their spaces) is an ongoing dynamic that requires daily attention, and whose conclusion is still unclear, which makes the history of the present so fascinating.

**Figure 4:**
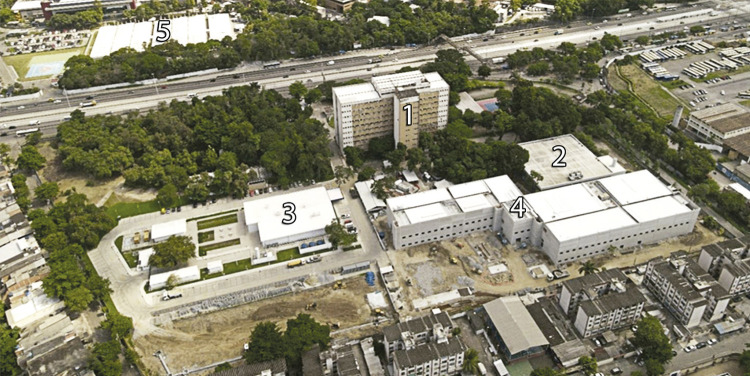
: Aerial image of the Maré campus in its concluding phase (Fiocruz Presidency’s archive)

## Final considerations

Twenty years after the publication of the book *Um lugar para a ciência: a formação do campus de Manguinhos [A Place for Science: the Shaping of the Manguinhos Campus]* (Oliveira, Costa, Pessoa, 2003), the return to this topic was not only an update to what was previously researched and published but also generated a new analysis of what was produced, the challenges that were faced, and the findings that were achieved. This article offers a firsthand look at the results of the continuation of this study, systematizing maps and data developed up to that time, and awaits more complete publication in book form, as was done in 2003.

The analysis of the past two decades of the history of the Oswaldo Cruz Foundation with regard to the construction choices selected for the Manguinhos-Maré campuses offers knowledge about institutional politics, but also set us against a series of challenges in writing about this context, since we are both subjects and agents of the time in which we live. As contemporaries, we find ourselves in the challenging situation described by Agamben (2009 , p.65), who calls attention to the fact that being contemporary “... means being able not only to firmly fix your gaze on the darkness of the epoch, but also to perceive in this darkness a light that, while directed toward us, infinitely distances itself from us.” As we understand Agamben, this light means revealing knowledge about a certain reality that in contemporaneity seems to us to escape every moment, even making it difficult to define a temporal demarcation.

In addition to this difficulty establishing temporal limits to address the present, reflections from the research presented in this article also bring with them challenges for history. In our analysis of the constructions on the campus, we were in line with the main discussions in a historiographic field, the present time, which allowed us to understand it from an analysis of various events that took place at the same time as our research.

The recent history of Fiocruz we have described here, with emphasis on the two objects analyzed in this study, is still under construction. As we have seen, the Health Technology Development Center (which was planned in the late 1980s and has been in the building phase since the 2000s) involved a series of negotiations and changes to the project and its management. Its conclusion is expected soon, along with the delivery of the group of buildings planned for the Maré campus. The latter case is a more recent endeavor that involves the institution’s position in response to the covid-19 pandemic, difficulties building in its original area (which has become increasingly dense in recent years and involves environmental and heritage protection issues), as well as its expansion area, which is changing the dynamic for the local community.

In this sense, talking about the present time involves many more challenges without immediate answers than we imagine we might encounter if we analyzed a “distant” historic period, since many of the events and facts analyzed herein took place simultaneously with our research and have not yet reached their conclusion. In the face of so many possibilities and “uncertainties” we have emphasized in our analysis, we conclude that we are in the middle of a dialectic process that involves observing the facts and the possibility of constructing them within a reading of the present time.

## NOTES

1 Study conducted at the Casa de Oswaldo Cruz/Fiocruz Department of Historical Heritage since June 2021, funded by the Fiocruz Institutional Development Incentive Program. The working group includes the researchers Renato da Gama-Rosa Costa, Inês El-Jaick Andrade, Renata Soares C. Santos, Matheus Gonçalves Góes, and Eric Gallo.

2 In this and other citations of texts from Portuguese, a free translation has been provided.

3 Record sheets are being produced for each building to gather the data collected during the study.

4 The former Dirac was transformed into Cogic in Decree 8932 of December 14, 2016. “In approving the Bylaws and the Chart Demonstrating Committee Positions and Confidence Functions in the Oswaldo Cruz Foundation (Fiocruz), changes have been made to the nomenclature of the units that cover Dirac and the other directorates.” See: https://www.cogic.fiocruz.br/pagina-exemplo/historico/ .

5 On the history of this hospital, see Benchimol (1990) ; Martire (2018) ; Santos (2019) ; Benchimol and Santos (2019) .

6 The original plan was developed by the Projeto Arquitetos Associados Ltda (Paal) company in 2004-2005 after Fiocruz held a public bid process for the project.

7 For more on the *Annales* movement, see Burke (1997) .

8 On the territorial demarcation of Fiocruz over its history, see Oliveira, Costa, Pessoa (2003); Benchimol (1990) .

9 The scientist and researcher Nísia da Trindade Lima was nominated by President Luíz Inácio Lula da Silva to be minister of Health, and assumed this post on January 2, 2023; at that time she was president of the Oswaldo Cruz Foundation (2017-2022).

## References

[B1] AGAMBEN Giorgio (2009). O que é o contemporâneo? E outros ensaios.

[B2] AREND Silvia Maria Fávero, MACEDO Fábio (2009). Sobre a história do tempo presente: entrevista com o historiador Henry Rousso. Revista Tempo e Argumento.

[B3] BÉDARIDA François, Ferreira Marieta de M., Amado Janaína (2006). Usos e abusos da história oral.

[B4] BENCHIMOL Jaime L (1990). Manguinhos do sonho à vida: a ciência na Belle Époque.

[B5] BENCHIMOL Jaime L., SANTOS Renata S.C., Amora Ana M.G.A., Costa Renato Gama-Rosa (2019). A modernidade na arquitetura hospitalar: contribuições para a historiografia.

[B6] BLOCH Marc (2001). Apologia da história: ou o ofício de historiador.

[B7] BRASIL Portaria SEDDM/SPU/ME Nº 11.521, de 23 de setembro de 2021. 23 set. 2021.

[B8] BURKE Peter (1997). A Escola dos Annales (1929-1989): a Revolução Francesa da historiografia.

[B9] COSTA Renato Gama-Rosa, SANTOS Renata Soares C., MARTIRE Giovanna (2022). ESHS Book of: Abstracts.

[B10] COSTA Rodrigo das Neves (2022). Entrevistadores: Renato da Gama-Rosa Costa, Inês El-Jaick Andrade, Renata Soares C. Santos, Matheus Góes, Éric Gallo.

[B11] COSTA Rodrigo das Neves (2019). Debaixo do mesmo teto. Prática projetual em edifícios de pesquisa e desenvolvimento biotecnológico.

[B12] DELGADO Lucília de Almeida Neves, FERREIRA Marieta de Moraes (2013). História do tempo presente e ensino de história. Revista História Hoje.

[B13] DO CRB-SAÚDE, Fiocruz ao Biobanco Covid-19 da Fiocruz Fiocruz.

[B14] DOSSE François (2009). L’Histoire à l’épreuve de la guerre des mémoires. Tempo e Argumento.

[B15] FERREIRA Marieta M (2018). Notas iniciais sobre a história do tempo presente e a historiografia no Brasil. Revista Tempo e Argumento.

[B16] FERREIRA Marieta M (2000). História do Tempo Presente: desafios. Cultura Vozes.

[B17] FIOCRUZ (2016). Relatório final. Congresso Interno da Fundação Oswaldo Cruz, 7., Conhecimento e inovação para a saúde, o desenvolvimento e a cidadania: o Estatuto da Fiocruz.

[B18] FIOCRUZ INAUGURA Biobanco Covid-19 (2021). Fiocruz.

[B19] FIOCRUZ INICIA construção de centro de pesquisa no campus Maré/Expansão (2021). Fiocruz.

[B20] GALLER Ricardo (2021). Entrevista de história oral, 2005.

[B21] GOOGLE EARTH (2022). Manguinhos.

[B22] HARLEY John Brian (2009). Mapas, saber e poder. Confins, Revista Franco-Brasileira de Geografia.

[B23] HARLEY John Brian (2005). La nueva natureza de los mapas.

[B24] HOBSBAWM Eric J, Hobsbawm Eric J (2004). Sobre história.

[B25] HOBSBAWM Eric J, Institut d’Histoire du Temps Présent (1993). Ecrire l’histoire du temps présent.

[B26] KAELBLE Hartmut, Institut d’Histoire du Temps Présent (1993). Ecrire l’histoire du temps présent.

[B27] LE GOFF Jacques (1990). História e memória.

[B28] LOHN Reinaldo Lindolfo, CAMPOS Emerson César de (2017). Tempo presente: entre operações e tramas. Revista História e Historiografia.

[B29] MARTIRE Giovanna Ermida (2018). Hospital Evandro Chagas: uma análise das transformações no edifício e diretrizes para o uso e ocupação.

[B30] MOREL Carlos Médicis, Centro de Desenvolvimento Tecnológico em Saúde (CDTS): um projeto estruturante da Fiocruz. (2019). Aula inaugural e abertura do ano letivo do Programa de Pós-graduação em Vigilância Sanitária do Instituto Nacional de Controle de Qualidade em Saúde/Fiocruz.

[B31] OLIVEIRA Benedito Tadeu de, COSTA Renato da Gama-Rosa, PESSOA Alexandre José de Souza (2003). Um lugar para a ciência: a formação do campus de Manguinhos.

[B32] OLIVEIRA Fabiano Arndt Araújo Pykosz de (2020). Os mapas contam histórias: reflexões, análises e perspectivas da utilização da cartografia histórica no ensino de história.

[B33] PREFEITURA da cidade do Rio de Janeiro (2013). Levantamento Aerofotogramétrico Digital do Rio de Janeiro. Folha 287-A-I-1. Escala 1:2.000.

[B34] PREFEITURA da cidade do Rio de Janeiro (1999). Levantamento Aerofotogramétrico Digital do Rio de Janeiro. Folha 287A. Escala 1:10.000.

[B35] RELATÓRIO FINAL da 2ª plenária extraordinária (2012). Sexto Congresso Interno da Fiocruz.

[B36] RICOEUR Paul (2007). A memória, a história, o esquecimento.

[B37] RICOEUR Paul (1997). Tempo e narrativa.

[B38] ROUSSO Henry, Ferreira Marieta de Moraes, Amado Janaína (2006). Usos e abusos da história oral.

[B39] SANTOS Renata Soares C (2019). O Instituto Oswaldo Cruz e seus hospitais: médicos, pacientes e suas mazelas rurais e urbanas (1909-1930).

